# On the Question of the Formation of Nitro-Functionalized 2,4-Pyrazole Analogs on the Basis of Nitrylimine Molecular Systems and 3,3,3-Trichloro-1-Nitroprop-1-Ene

**DOI:** 10.3390/molecules27238409

**Published:** 2022-12-01

**Authors:** Karolina Kula, Agnieszka Łapczuk, Mikołaj Sadowski, Jowita Kras, Karolina Zawadzińska, Oleg M. Demchuk, Gajendra Kumar Gaurav, Aneta Wróblewska, Radomir Jasiński

**Affiliations:** 1 Department of Organic Chemistry and Technology, Cracow University of Technology, Warszawska 24, 31-155 Krakow, Poland; 2 Faculty of Medicine, The John Paul II Catholic University of Lublin, Konstantynow 1J, 20-708 Lublin, Poland; 3 Sustainable Process Integration Laboratory—SPIL, NETME Centre, Faculty of Mechanical Engineering, Brno University of Technology—VUT Brno, Technická 2896/2, 616-69 Brno, Czech Republic; 4 Centre of Molecular and Macromolecular Studies, Polish Academy of Sciences, Sienkiewicza 112, 90-363 Lodz, Poland

**Keywords:** pyrazoline, pyrazole, nitrocompound, nitrylimine, cycloaddition reaction, molecular electron density theory, synthesis

## Abstract

Experimental and theoretical studies on the reaction between (*E*)-3,3,3-trichloro-1-nitroprop-1-ene and *N*-(4-bromophenyl)-C-arylnitrylimine were performed. It was found that the title process unexpectedly led to 1-(4-bromophenyl)-3-phenyl-5-nitropyrazole instead of the expected Δ^2^-pyrazoline molecular system. This was the result of a unique CHCl_3_ elimination process. The observed mechanism of transformation was explained in the framework of the molecular electron density theory (MEDT). The theoretical results showed that both of the possible channels of [3 + 2] cycloaddition were favorable from a kinetic point of view, due to which the creation of 1-(4-bromophenyl)-3-aryl-4-tricholomethyl-5-nitro-Δ^2^-pyrazoline was more probable. On the other hand, according to the experimental data, the presented reactions occurred with full regioselectivity.

## 1. Introduction

Pyrazolines are heterocyclic systems containing nitrogen in their structure. They are significant organic compounds with many applications, not only as components for further organic synthesis but also as complete products. Pyrazolines and their analogs are five-membered heterocyclic compounds with one unsaturated double bond and two adjacent nitrogen atoms [1,2,3], which make them readily undergo various transformations. Due to the presence of the unsaturated bond and nitrogen atoms, pyrazolines have great biological activity and have many properties related to this. Many of these compounds are widely used in medicine as well as in other departments of industry. In particular, pyrazoline analogs show anticancer [4], anti-inflammatory [5], antiobesity, analgesic and antidepressant properties [6,7]. These compounds are also effective in treating Alzheimer’s disease [8]. What is more, there are many drugs based on pyrazoline’s structure, e.g., celecoxib [9], rimonabant, difenamizole and fezolamine. In industry, pyrazolines have been known since the early 1970s [10,11,12]. They were successfully applied as insecticides but also as inhibitors that control sodium channel functions [13,14]. Furthermore, the pyrazoline ring can be found in a variety of pesticides, mainly in fungicides [15,16], insecticides [17] and herbicides, including patented products such as fenpyroximate [18], fipronil [19,20], tebufenpyrad [21] and tolfenpyrad [20].

At this moment, several methods of pyrazoline preparation are known. The most common include the cyclocondensation of α,β-unsaturated carbonyl compounds with hydrazine; the 1,3-alkylation reaction of hydrazines with 1,3-dihalopropanes; the intramolecular cyclization of hydrazones; as well as [3 + 2] the cycloaddition reaction (32CA) of alkene with diazocompounds, nitrylimines and also azomethine imines [22].

**Scheme 1:**
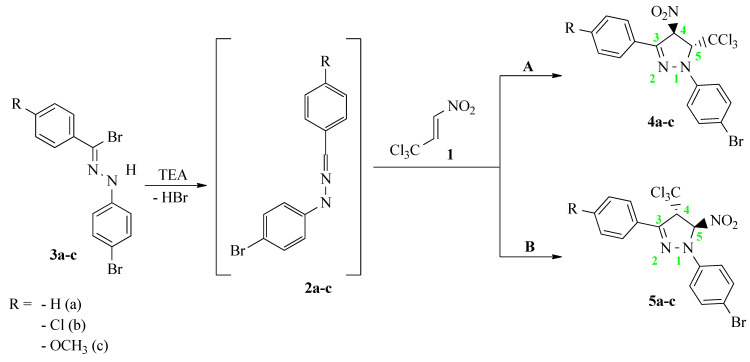
Theoretically possible reaction paths of 32CA between TNP (**1**) and NIs (**2a**–**c**).

In the framework of this paper, we decided to shed a light on the possibility of the synthesis of new nitro-substituted analogs of pyrazoline on the basis of a 32CA reaction [23]. For the model of nitroalkene, we selected (*E*)-3,3,3-trichloro-1-nitroprop-1-ene (TNP) (**1**). TNP can be characterized by a very reactive structure for organic synthesis and the ability to insert different CX_3_ and NO_2_ groups into the main product, which additionally stimulates many important biologically active functions [24,25,26,27,28]. Moreover, this nitroalkene was successfully tested in 32CA reactions with many organic compounds, such as nitrones [29,30,31], nitrile *N*-oxides [32,33], diazocompounds [2,3,34] and *N*-azomethine ylides [35,36,37], and in other reactions [38,39,40]. In turn, to model the three-atom component (TAC) [23], we decided to use *N*-(4-bromophenyl)-C-phenylnitrylimine (NI) (**2a**). For such defined addends, reactions can be theoretically realized with two regioisomeric paths, leading to cycloadducts with a nitro group at the fourth (**4a**) or fifth (**5a**) position of a ring (Scheme 1).

## 2. Result and Discussion

In the first part of our research, we decided to shed a light on the nature of the interactions between (*E*)-3,3,3-trichloro-1-nitroprop-1-ene (**1**) and *N*-(4-bromophenyl)-C-phenylnitryl-imine (**2a**) and (on the margins of the main stream of the research) its substituted analogs (**2b**,**c**) (Scheme 1). For this purpose, the approach in the framework of the molecular electron density theory (MEDT) [41] was applied. Therefore, we calculated the respective global and local electronic properties of the model reaction components using conceptual density functional theory (CDFT) [42,43] methodologies. The CDFT is known as a strong implement that helps predict the reactivity of the components in polar processes such as cycloaddition. According to Domingo recommendations, all of the indices were determined via calculations based at the B3LYP/6-31G(d) level of theory in the gas phase [42,43,44]. The global reactivity indices, namely the electronic chemical potential μ, chemical hardness η, global electrophilicity ω and global nucleophilicity N, were determined and given in Table 1.

The value of the electronic chemical potential [42] μ can estimate the direction of the electron density flux between reagents in the determined path of the reaction. In the case of the reaction between (*E*)-3,3,3-trichloro-1-nitroprop-1-ene (**1**) and *N*-(4-bromophenyl)-C-phenylnitryl-imine (**2a**), the electronic chemical potentials μ were equal to −5.84 eV and −3.32 eV, respectively (Table 1). This indicated that in the course of this reaction, the flux of the electron density would take place in the conversion from nitrylimine (**2a**) to nitroalkene (**1**). Analogously, equal results could be observed for the other reagent systems, as, in all cases, nitrylimine (**2b**–**c**) was characterized by a higher value of the electronic chemical potential than nitroalkene (**1**). To sum up, all presented cycloaddition reactions should be classified as forward electron density fluxes (FEDF) in agreement with the CDFT analysis [45,46,47,48].

Next, the calculated values were global indices of the electrophilicity [44] ω and nucleophilicity [49] N of the reagents. The electrophilicity ω indices of nitrylimines (**2a**–**c**) fell within a range from 1.43 eV to 1.84 eV (Table 1), which allowed the conclusion that these compounds act like moderate electrophiles in a polar reaction due to the electrophilicity scale [42,50]. In turn, the nucleophilicity N indices of nitrylimines **2a**–**c** equaled from 3.88 eV to 3.86 eV, respectively (Table 1), which allowed the characterization of nitrylimines (**2a**–**c**) as rather strong nucleophiles in polar reactions, which was in agreement with the nucleophilicity scale [42,50].

Both the presence of an ER group, such as –OCH_3_, or an EW group, such as –Cl, at the fourth position of the phenyl ring in nitrylimines (**2a**–**c**) insignificantly changed the global electronic properties of these TAC. On the other hand, (*E*)-3,3,3-trichloro-1-nitroprop-1-ene (**1**) was characterized by an electrophilicity index ω of 3.27 eV and a nucleophilicity index N of 0.66 eV (Table 1). These values allowed the categorization of nitroalkene (**1**) as a super-strong electrophile and as a marginal nucleophile in polar reactions within the electrophilicity and nucleophilicity scales [42,50].

To be able to consider this reaction as a polar process, the character of the components need to be determined. Polar processes occur between good electrophiles and good nucleophiles. According to CDFT calculations (Table 1), the analyzed reactions between (*E*)-3,3,3-trichloro-1-nitroprop-1-ene (**1**) and nitrylimines (**2a**–**c**) are characterized by the major difference in their electrophilicity index (Δω > 1 eV) [51,52]. According to this, the presented reactions should be classified as polar processes.

**Figure 1:**
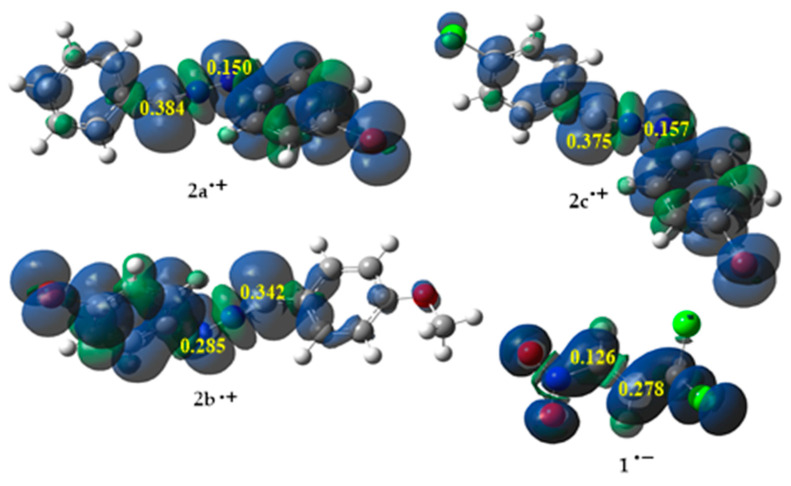
The local electronic properties of substrates **1** and **2a**–**c** as three-dimensional representations (3D) of Mulliken atomic spin densities for the nitroalkene **1˙ˉ** radical anion and the nitrylimines **2a**–**c˙^+^** radical cations, together with values of the electrophilic (P_k_^+^) Parr function of **1** and the nucleophilic (P_k_^−^) Parr functions of **2a**–**c**.

The regioselectivity of the polar process in which nonsymmetric reagents take part can be easily determined by measuring the interactions between the most electrophilic center, located in the electrophile, and the most nucleophilic center, located in the nucleophile. The most exact way to describe local reactivity is through electrophilic (radical-anionic) Parr functions P_k_^+^ and nucleophilic (radical-cationic) Parr functions P_k_^−^ [52,53,54,55]. To determine the abovementioned centers of the components, the Parr functions for the reagents were calculated and given in Figure 1.

The analysis of the electrophilic Parr function P_k_^+^ of (*E*)-3,3,3-trichloro-1-nitroprop-1-ene (**1**) showed that the most electrophilic center was located on the Cβ carbon atom, P_Cβ_^+^ = 0.278, which means that this center should react with the most nucleophilic center of substituted nitrylimines. In turn, the results of the nucleophilic Parr functions P_k_^−^ for nitrylimines (**2a**–**c**) showed that the most nucleophilic center was located on the carbon atom of the –N=N=**C**-structure fragment. Both of the presented centers determined the most favorable path of the considered reactions to be path **B**, leading to the products **5a**–**c** (Scheme 1) [52,55].

Next, we searched for good conditions for the reaction involving (*E*)-3,3,3-trichloro-1-nitroprop-1-ene (**1**) and *N*-(4-bromophenyl)-C-phenylnitrylimine (**2a**). For this purpose, we conducted several tests using different reaction conditions, solvents and temperatures (Table 2). It was found that the analyzed process proceeded easily in benzene solution at room temperature. The TLC and HPLC examination of the postreaction mixture showed, without any doubts, the presence of only one reaction product (R_f_ = 0.56 and R_t_ = 6.1 min, respectively). It was easily isolated through column chromatography (see experimental part), and a solid material characterized by its adequate purity for analysis was obtained. 

**Scheme 2:**
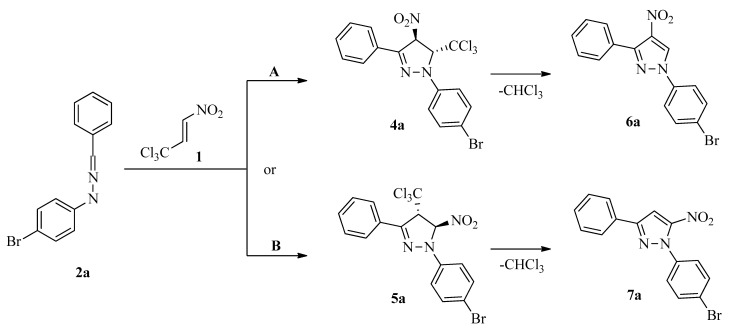
Competitive paths for the formation of regioisomeric pyrazoles (**6a** or **7a**).

The IR spectrum of the isolated compound confirmed the presence of =N-N< and >C=N- moieties as well as the NO_2_ group [56]. Unexpectedly, no bands connected with the expected existence of C-Cl bonds were detected. Next, based on HRMS analysis, the compound was assigned the molecular formula of C_15_H_10_N_3_O_2_Br. The molecular weight of its molecular ion ([M + H]^+^ = 344.0029) was about 119.5 lower than those of the expected [3 + 2] cycloadducts. Lastly, in the ^1^H-NMR spectrum, the pair of expected doublets connected with the protons in the NO_2_-C(H)-C(H)-CCl_3_ moiety was not detected. Due to the facts mentioned above, the isolated compound was not the expected pyrazoline (**4a** or **5a**) but a product (**6a** or **7a**) of the chloroform extrusion of the primary cycloadduct (**4a** or **5a**) (Scheme 2).

Unfortunately, the regioisomerism of the obtained pyrazole system was disputable because all the attempts to obtain a crystal form for RTG structural analysis were not successful. On the other hand, some valuable information regarding adduct regiochemistry can be derived from the NMR experiments. In particular, independently of the signal of the proton in the heterocyclic ring in the ^1^H NMR spectrum, we precisely identified signals from two different substituted benzene rings on the ^1^H and ^13^C NMR spectra (see Supplementary Materials). Using the HMBC experiment (see Supplementary Materials), we detected a clear correlation between the signal derived from the carbon atom of the benzene ring at the third position of the pyrazole molecular system and the signal derived from the proton connected directly to the pyrazole molecular system. On the other hand, similar correlations between the same proton and carbon atoms from the 4-bromophenyl moiety were not observed. This confirmed that the nitro group must be located at the C5 position of heterocyclic ring and, as a consequence, clearly showed that the isolated product exhibited the constitution of 1-(4-bromophenyl)-3-phenyl-5-nitropyrazole **7a**. Therefore, as the primary [3 + 2] cycloaddition product, the adduct **5a** should be exclusively considered.

**Figure 2:**
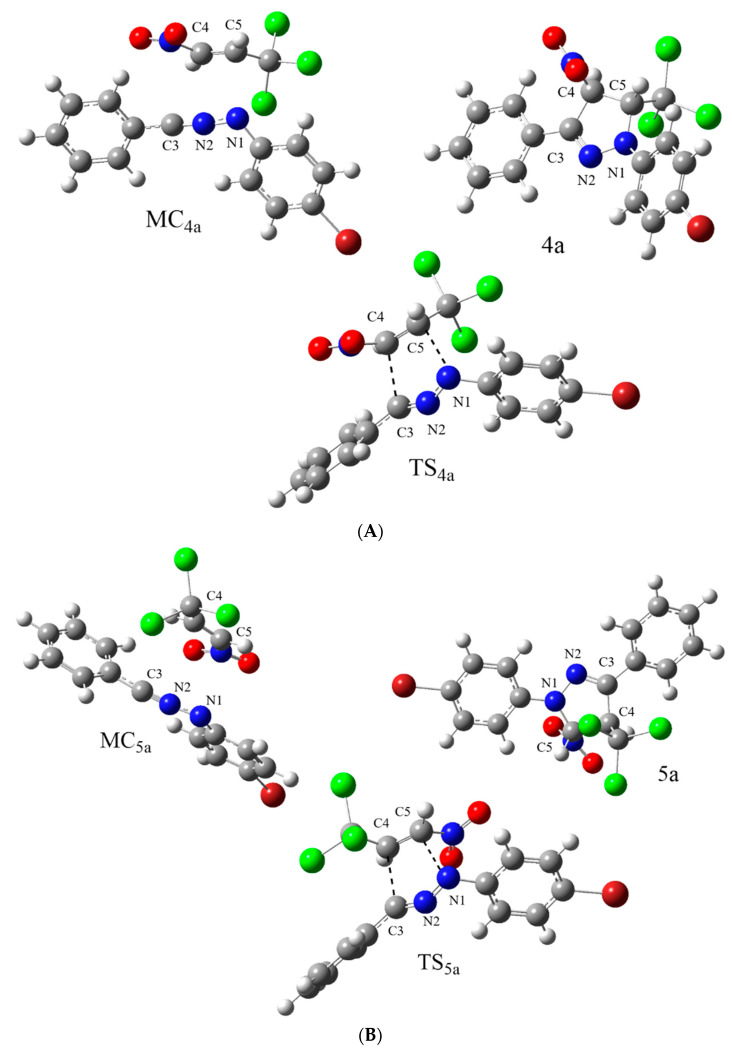
Views of critical structures of 32CA reaction of TNP (**1**) with NI (**2a**) in benzene solution according to WB97XD/6-311G(d,p)(PCM) calculations, for pathways (**A**) and (**B**) (Scheme 1).

In the next step, we decided to shed some light on the **1 + 2a** cycloaddition process course using the DFT exploration of theoretically possible reaction channels (Scheme 2). It was found that both possible reaction channels were very similar in their nature. In particular, the first stage was the formation of a molecular complex (**MC**) (Figure 2). This was accompanied by the decrease of the enthalpy of the reaction system by a few kcal/mol. Due to the entropic factor, the Gibbs free energies of the formation of MCs were positive. This excluded the possibility of the existence of MCs as stable intermediates. Within MCs, no new chemical bonds were formed. Similar-type intermediates have been identified recently regarding many bimolecular processes, such as 32CAs [32,57,58,59], Diels–Alder reactions [54,60,61] and others [62,63,64]. Independent of the considered cycloaddition channel, the second reaction stage was a formation of the transition-state structure (**TS**) (Figure 2). The energetic barriers connected to the existence of TSs were relatively low (Table 3).

It was found interesting that the formation of pyrazoline **5a** was evidently favored from a kinetic point of view. This observation correlated well with the regiochemistry observed experimentally. Within TSs, the key interatomic distances were substantially reduced (Table 4). The synchronicity of the new bond formations was similar regarding both of the analyzed TSs. Next, the global electron density transfer (GEDT) [65] values suggested that optimized TSs should be treated as polar structures. However, this polarity was not sufficient in enforcing the stepwise, zwitterionic mechanism [66]. In a similar manner, we also theoretically explored 32CAs involving other nitrilimines (**2b**,**c**) (see Supplementary Materials). Our research suggested similar reaction regioselectivities and molecular mechanisms as well as the similar nature of their critical structures. Therefore, the suggested protocol for the preparation of nitro-substituted pyrazole analogs can be treated as a general protocol in some range.

## 3. Materials and Methods

### 3.1. Materials

Commercially available (Sigma–Aldrich, Szelągowska 30, 61-626 Poznań, Poland) reagents and solvents were used. All solvents were tested with high pressure liquid chromatography before use. (*E*)-3,3,3-trichloro-1-nitroprop-1-ene (**1**) and *N*-(4-bromophenyl)-C-phenylnitrylimine (**2a**) were prepared in reactions described in literature [67,68,69].

### 3.2. General Procedure for Cycloaddition Reactions

A mixture of 10 mmol of *N*-(4-bromophenyl)-*N*’-benzylidenehydrazine bromide (**1a**); 20 mmol of (*E*)-3,3,3-trichloro-1-nitroprop-1-ene (**3**); and 10 mmol of triethylamine in 5 mL of dry benzene was stirred in the dark at room temperature for 6 h. After that, the post-reaction mixture was washed with water, and solvent was evaporated in vacuo. The obtained semisolid mass was washed with cold petroleum ether, purified using column chromatography (stationary phase: SiO_2_, mobile phase: C-hex:AcOEt 9:1) and crystallized from ethanol. A white amorphous solid was obtained.

1-(4-bromophenyl)-3-phenyl-5-nitropyrazole (**7a**) was obtained as C_15_H_10_N_3_O_2_Br. Its yield was 93%, and its m.p. was 189–194 °C (EtOH). IR (KBr) results were as follows: υ was 1617 (>C=C<), 1545 and 1351 (NO_2_), 1402 (=N-N<) and 1280 (-N=C<) cm^−1^. UV-Vis (MeOH) results were as follows: λ was 262 nm. ^1^H NMR (500 MHz, CDCl_3_) results were as follows: δ was 8.76 (s, 1H, CH=C–NO_2_), 7.80–7.77 (m, 2H, CH_Ar_), 7.70–7.66 (m, 4H, CH_Ar_) and 7.52–7.49 (m, 3H, CH_Ar_). ^13^C NMR (125 MHz, CDCl_3_) results were as follows: δ was 148.53; 144.59; 137.46; 132.95; 129.78; 129.45; 128.26; 128.11; 122.24; 121.04; and 100.30. HR-MS (ESI, 200 °C) results calculated for C_15_H_10_N_3_O_2_Br [M + H]^+^ = 344.0019 with a found value of 344.0029.

### 3.3. Analytical Techniques

For reaction progress testing, liquid chromatography (HPLC) was performed using a KNAUER apparatus equipped with a UV-Vis detector with application of the standard procedure [70,71,72]. LiChrospher RP-18 10 μm column (4 × 250 mm) was applied and methanol–water MeOH:H_2_O (70:30 *v*/*v*) was used as eluent at a flow rate of 1.5 cm^3^ min^−1^. Melting points were determined with the Boetius PHMK 05 apparatus and were not corrected. IR spectra (KBr pellets) were registered on a Thermo Nicolet 6700 FT-IR apparatus. ^1^H NMR (500 MHz) and ^13^C NMR (125 MHz) spectra were recorded with a Bruker AVANCE NMR spectrometer using CDCl_3_ as a solvent. TMS was used as an internal standard. UV-Vis spectra were determined for the 200–500 nm range through usage of spectrometer UV-5100 Biosens. HR-MS spectra were performed on a Shimadzu LCMS-IT-TOF instrument with ES ionization (heat bock and CDL temperatures of 200 °C, nebulizing gas flow of 1.5 mL/min) connected to a Shimadzu Prominence two LC-20AD pump chromatograph equipped with Phenomenex Kinetex 2.6 µm C18 100A column (acetonitrile–water mixtures, ACN:H_2_O, 65:35 *v*/*v,* were used as the eluent).

### 3.4. Computational Details

All computations were performed using the Gaussian 09 package [73] in the Prometheus computer cluster of the CYFRONET regional computer center in Cracow. DFT calculations were performed using the WB97XD/6-311G(d,p) [74,75] level of theory. A similar computational level has already been successfully used for the exploration of mechanistic aspects of other cycloaddition processes [45,46,47,76]. Calculations of all critical structures were performed at temperature T = 298 K and pressure *p* = 1 atm. All localized stationary points were characterized using vibrational analysis. It was found that starting molecules as well as products had positive Hessian matrices. On the other hand, all transition states showed only one negative eigenvalue in their Hessian matrices.

For all optimized transition states, intrinsic reaction coordinate (IRC) [77] computations were performed to verify that the located TSs were connected to the corresponding minimum stationary points associated with reactants and products. The solvent effects were simulated using a standard-procedure self-consistent reaction field (SCRF) [78,79] based on the polarizable continuum model (PCM) [80].

The global electron density transfer (GEDT) [68] values were designated based on the formula GEDT = |ΣqA|, where qA is the net Mulliken charge and where the sum is performed over all the atoms of (*E*)-3,3,3-trichloro-1-nitroprop-1-ene (**3**). In turn, the σ-bond development (l) indices were designated based on the formula [51,54]:

$$l_{X-Y}=1-\frac{r{\;}_{X-Y}^{TS}-r{\;}_{X-Y}^{P}}{r{\;}_{X-Y}^{P}}$$

where *r^TS^_X−Y_* is the distance between the reaction centers *X* and *Y* in the transition structure and where *r^P^_X−Y_* is the same distance in the corresponding product.

Global electronic properties of reactants, according to Domingo recommendations, were performed at B3LYP/6-31G(d) level of theory in the gas phase [42,43,44]. Electrophilic Parr functions P_k_^+^ and nucleophilic Parr functions P_k_⁻ were obtained from the changes in atomic spin density (ASD) of the reagents [52,55].

GaussView program [81] was used to visualize molecular geometries of all the systems as well as to show 3D representations of the radical anion and the radical cation.

## 4. Conclusions

The reaction between (*E*)-3,3,3-trichloro-1-nitroprop-1-ene and *N*-(4-bromophenyl)-C-arylnitrylimines was examined using experimental and theoretical methods. The theoretical results showed that both of the considered 32CA channels are possible from a kinetic point of view of which the formation of 1-(4-bromophenyl)-3-aryl-4-tricholomethyl-5-nitro-Δ^2^-pyrazoline is more probable. This conclusion was compatible with the presented MEDT study, where interactions between the Cβ atom of electrophilic (*E*)-3,3,3-trichloro-1-nitroprop-1-ene and the carbon atom of the –N=N=C- fragment of nucleophilic nitrilimine determine a more probable reaction path.

In turn, the experimental results of the 32CA reaction between (*E*)-3,3,3-trichloro-1-nitroprop-1-ene and *N*-(4-bromophenyl)-C-phenylnitrylimine showed that the presented reaction occurred with full regioselectivity. The obtained products were extremely unstable and spontaneously converted through CHCl_3_-elimination to pyrazole systems. As a result, 1-(4-bromophenyl)-3-phenyl-5-nitropyrazole was obtained.

## Figures and Tables

**Table 1:** Global electronic properties (electronic chemical potential μ, chemical hardness η, global electrophilicity ω and nucleophilicity N; all in eV) for the studied reagents.

Reagent	μ	η	ω	N
**1**	−5.84	5.22	3.27	0.66
**2a**	−3.32	3.84	1.66	3.76
**2b**	−3.51	3.70	1.84	3.86
**2c**	−3.55	3.42	1.43	3.88

**Table 2:** Selected examples of reactions between TNP (**1**) and NI (**2a**) under different conditions.

Temperature [°C]	Solvent	Reaction Time [h]	Catalyst	Yield [%]
25	Benzene	6	-	-
25	Benzene	24	-	-
25	Benzene	6	Et_3_N	93
80	Benzene	6	Et_3_N	65
25	Benzene	6	*n*-PrNH_2_	70
25	Benzene	6	CsCO_3_	Trace
80	Benzene	6	CsCO_3_	Trace

**Table 3:** Kinetic and thermodynamic parameters of 32CA reaction of TNP (**1**) with NI (**2a**) according to WB97XD/6-311G(d,p)(PCM) calculations (ΔH and ΔG are given in kcal·mol^−1^; ΔS is given in cal·mol^−1^ K^−1^).

Path	Transition	ΔH	ΔG	ΔS
**A**	**1 + 2a ** **→ MC_4a_**	−6.61	3.32	−42.41
	**1 + 2a ** **→ TS_4a_**	−0.74	11.52	−50.26
	**1 + 2a ** **→ 4a**	−64.91	−50.90	−56.14
**B**	**1 + 2a ** **→ MC_5a_**	−8.80	0.69	−40.97
	**1 + 2a ** **→ TS_5a_**	−2.25	10.50	−51.90
	**1 + 2a ** **→ 5a**	−67.06	−53.01	−56.26

**Table 4:** The key parameters of the critical structure parameters of 32CA reaction of TNP (**1**) with NI (**2a**) according to WB97XD/6-311G(d,p)(PCM) calculations.

	C3-C4 r [Å]	l_C3-C4_	C5-N1 r [Å]	l_C5-N1_	Δl	GEDT [e]
**MC_4a_**	3.260		3.033			0.03
**TS_4a_**	2.242	0.521	2.355	0.378	0.14	0.17
**4a**	1.516		1.452			
**MC_5a_**	3.333		3.033			0.03
**TS_5a_**	2.275	0.509	2.383	0.309	0.20	0.20
**5a**	1.526		1.409			

## Data Availability

The data presented in this study are available on request from the corresponding author.

## Supplementary Materials

The following supporting information can be downloaded at: https://www.mdpi.com/article/10.3390/molecules27238409/s1, Spectral characteristic, pp. S2–S6. Computational data, pp. S7–S14.

## Sample Availability

Samples of the compounds are available from the corresponding authors.
