# Supplementary material for: On the Question of the Formation of Nitro-Functionalized 2,4-Pyrazole Analogs on the Basis of Nitrylimine Molecular Systems and 3,3,3-Trichloro-1-Nitroprop-1-Ene
Source: Molecules. 2022 Dec 1;27(23):8409. doi: 10.3390/molecules27238409 (PMC9739753; doi:10.3390/molecules27238409)
Supplement: Supplementary file 1 [file molecules-27-08409-s001.zip › molecules-2071890-supplementary.pdf]

# SUPPLEMENTARY MATERIALS

# On the question of the formation nitro-functionalized 2,4-pyrazole analogs on the basis of nitrylimine molecular systems and (E)-3,3,3-trichloro-1-nitroprop-1-ene

**Karolina Kula [1,*], Agnieszka Łapczuk [1,*], Mikołaj Sadowski [1], Jowita Kras [1], Karolina Zawadzińska [1], Oleg M. Demchuk [2], Gajendra Kumar Gaurav [3], Aneta Wróblewska [4] and Radomir Jasiński [1,*]**

[1] Institute of Organic Chemistry and Technology, Cracow University of Technology, Warszawska 24, 31-155 Cracow, Poland;

[2] Faculty of Natural Sciences and Health, The John Paul II Catholic University of Lublin, Konstantynow 1H, 20-708 Lublin, Poland;

[3] Sustainable Process Integration Laboratory – SPIL, NETME Centre, Faculty of Mechanical Engineering, Brno University of Technology – VUT Brno,
Technická 2896/2, 616 69 Brno, Czech Republic;

[4] Centre of Molecular and Macromolecular Studies, Polish Academy of Sciences, Sienkiewicza 112, 90-363 Lodz, Poland;

* Correspondence adress: karolina.kula@pk.edu.pl (K.K)
agnieszka.lapczuk@pk.edu.pl (A.Ł.)
radomir.jasinski@pk.edu.pl (R.J.)

## PHYSICAL CHARACTERISTICS

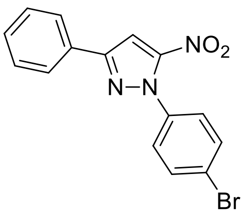


**1-(4-bromophenyl)-3-phenyl-5-nitropyrazole (7a)** $C_{15}H_{10}N_3O_2Br$.

White amorphous solid; yield: 93%; m.p. 189-194°C (MeOH); IR (KBr): υ: 1617 (>C=C<); 1545 and 1351 ($NO_2$); 1402 (=N-N<); 1280 (-N=C<) $cm^{-1}$; UV-Vis (MeOH): λ: 262 nm; $^{1}H$ NMR (500 MHz, $CDCl_3$): δ: 8.76 (s, 1H, C<u>H</u>$^{4}$); 7.80-7.77 (m, 2H, C<u>H</u>$^{3A}$); 7.70-7.66 (m, 4H, C<u>H</u>$^{1A}$+C<u>H</u>$^{1B}$); 7.52-7.49 (m, 3H, C<u>H</u>$^{3B}$+C<u>H</u>$^{3C}$); $^{13}C$ NMR (125 MHz, $CDCl_3$): δ: 148.53 (C4); 144.59 (C3); 137.46 (C6); 132.95 (C12); 129.78 (C17); 129.45 (C9+C10); 128.26 (C15+C16); 128.11 (C13+C14); 122.24 (C7+C8); 121.04 (C11); 100.30 (C5); HR-MS (ESI, 200°C): $[M+H]^+$ for $C_{15}H_{10}N_3O_2Br$ calculated 344.0019, found 344.0029;

## IR

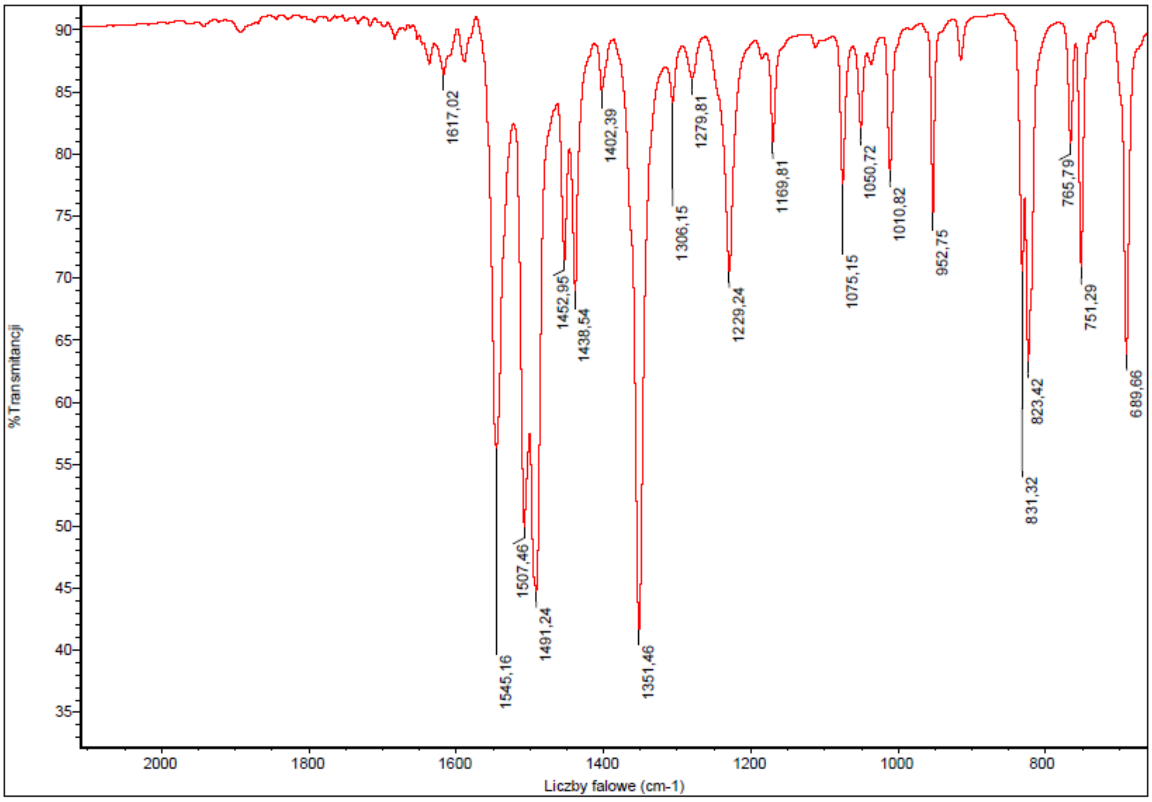


## HR-MS

| Rank | Score | Formula (M) | Ion | Meas. m/z | Pred. m/z | Diff (mDa) | Diff (ppm) | Iso Score | DBE |
|---|---|---|---|---|---|---|---|---|---|
| 1 | 63.19 | C15 H10 N3 O2 Br | [M+H] + | 344.0019 | 344.0029 | -1.0 | -2.91 | 66.36 | 12.0 |

MS Spectrum Graph

#:1 Ret.Time:Averaged 11.720-12.210(Scan#:4689-4885)
BG Mode:Averaged 13.530-17.500(5413-7001)
Mass Peaks:5 Base Peak:346.00(524790) MS Stage:MS Polarity:Pos Segment1 - Event1 Precursor:----- Cutoff: Ionization Mode:E

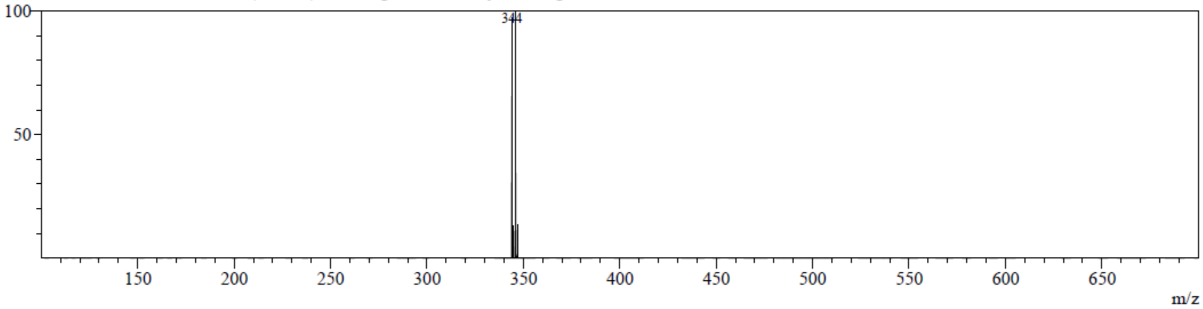

**¹H NMR**

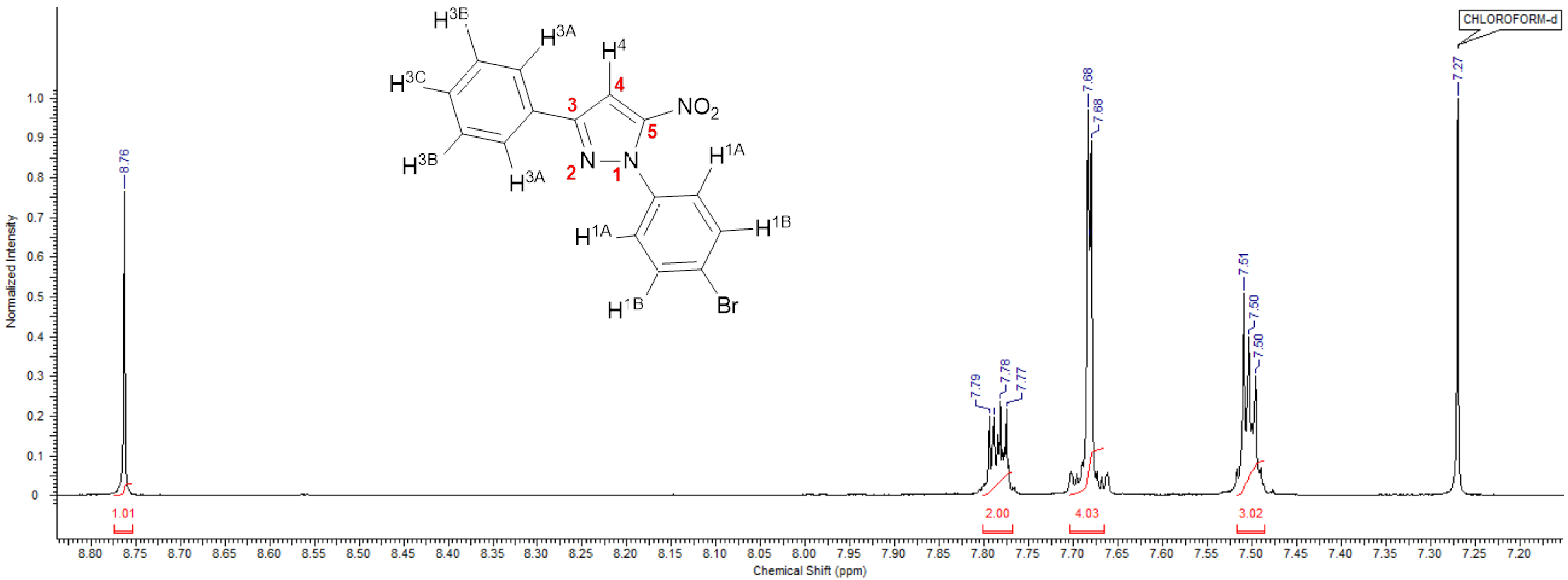

CHLOROFORM-d
8.76
7.79
7.78
7.77
7.68
7.68
7.51
7.50
7.50
7.27
1.01
2.00
4.03
3.02
Normalized Intensity
Chemical Shift (ppm)
H3B
H3A
H4
H3C
NO2
H1A
H1B
Br

$^{13}C$ NMR

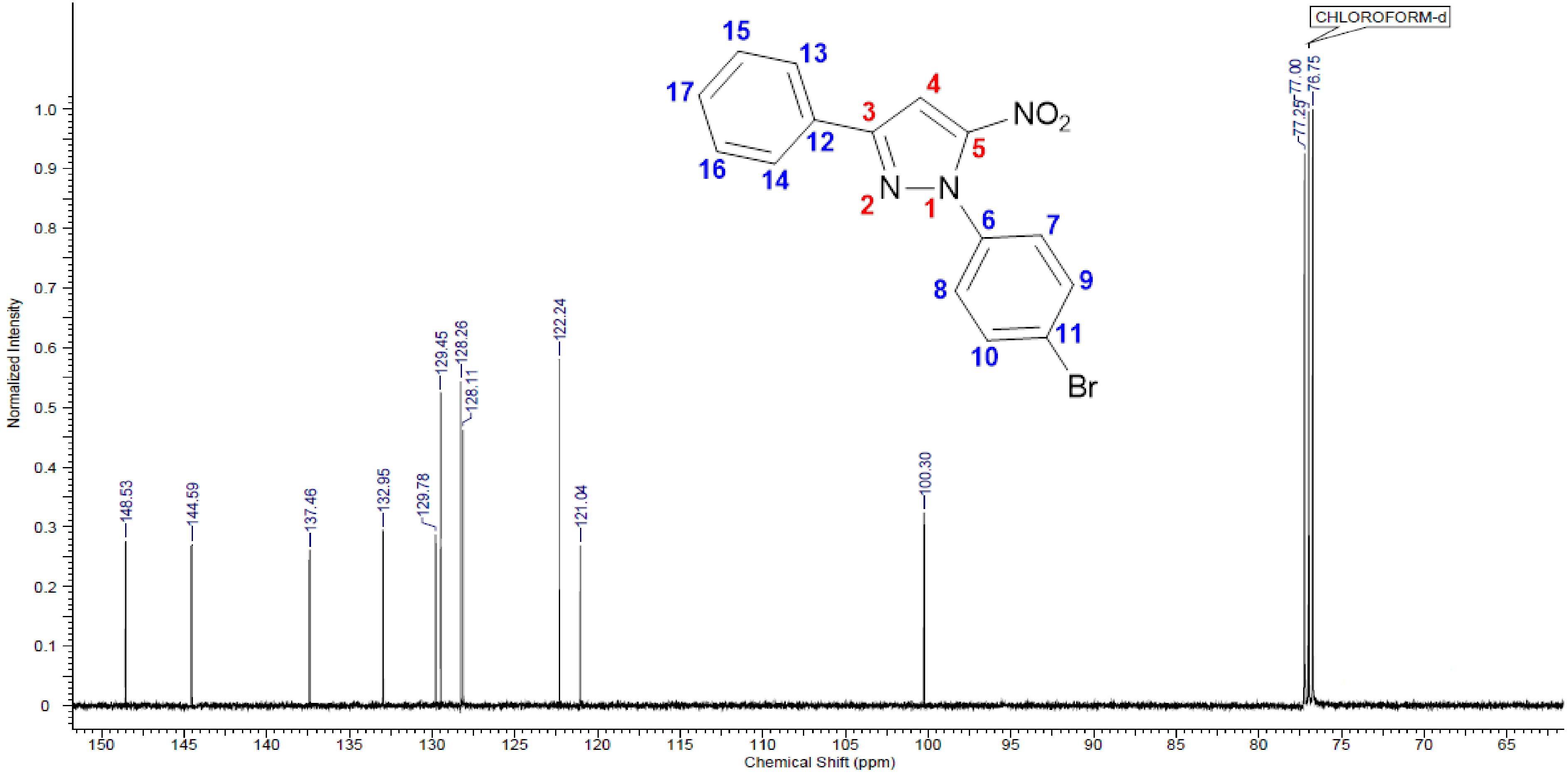
CHLOROFORM-d
148.53
144.59
137.46
132.95
129.78
129.45
128.26
128.11
122.24
121.04
100.30
77.25
77.00
76.75
NO2
Br
Normalized Intensity
Chemical Shift (ppm)

**HMBC**

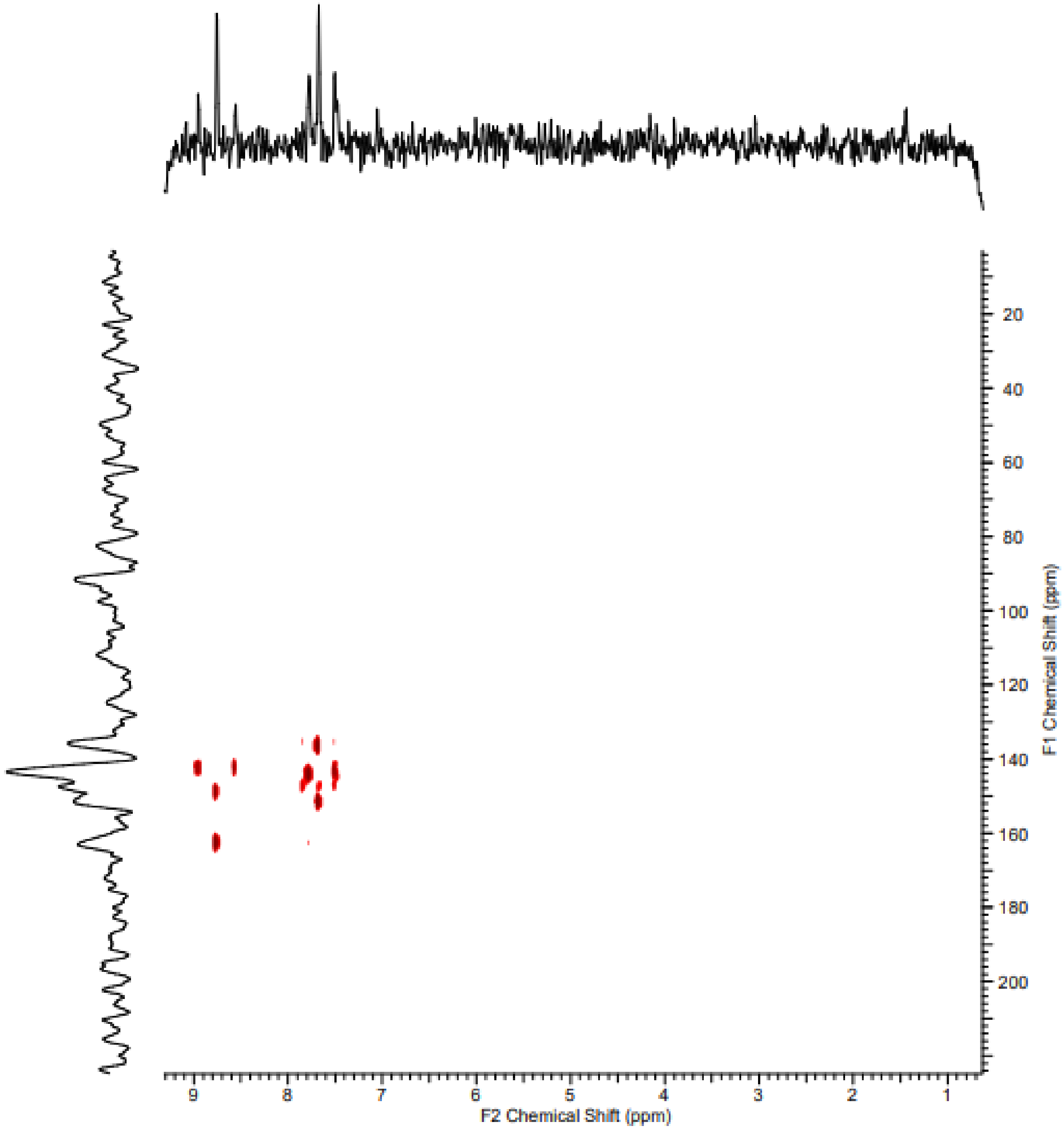

20
40
60
80
100
120
140
160
180
200
F1 Chemical Shift (ppm)
9
8
7
6
5
4
3
2
1
F2 Chemical Shift (ppm)

Fragment of HMBC spectrum

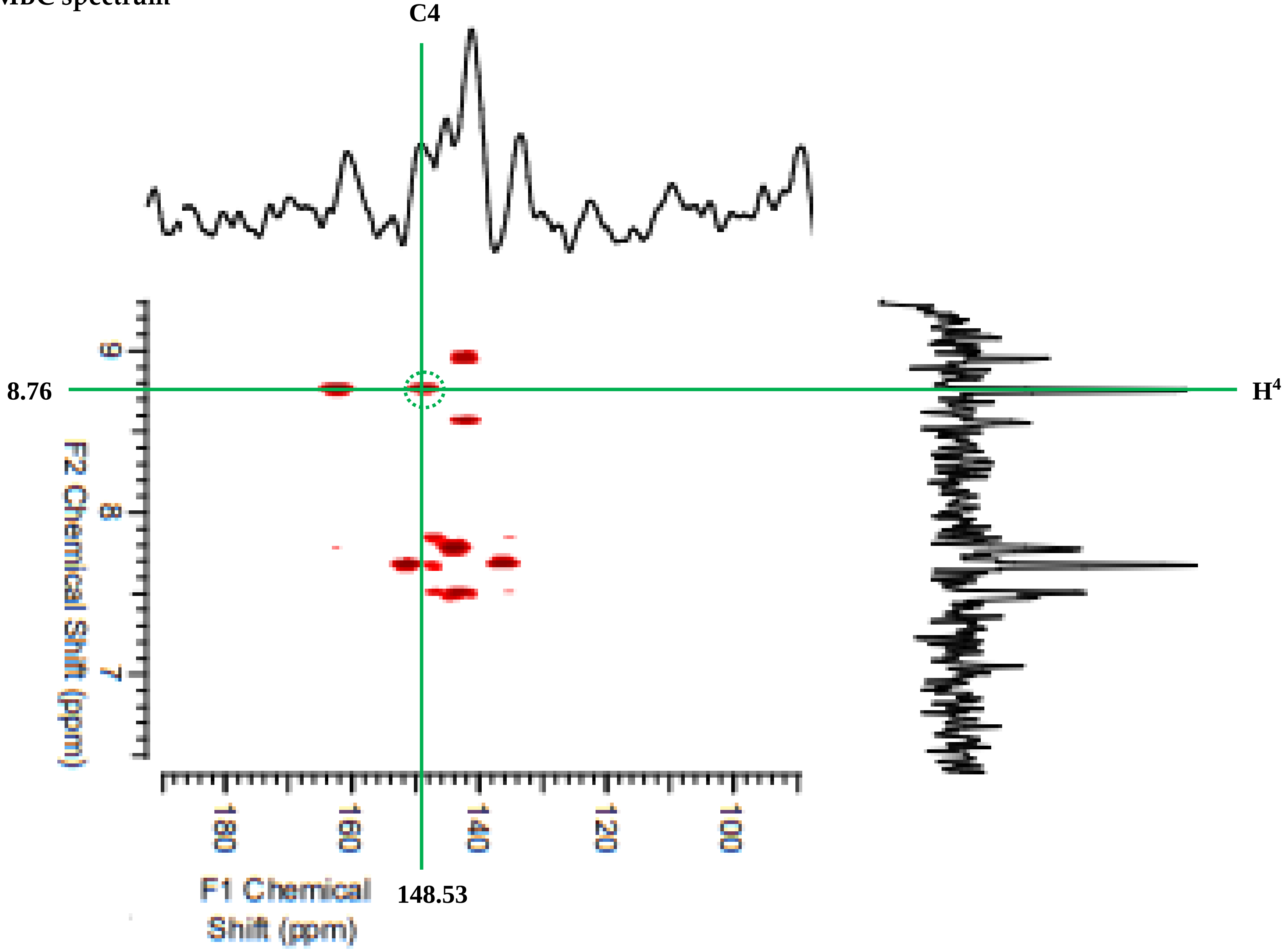

**Table S1.** Kinetic and thermodynamic parameters for 32CA reaction of TNP (**1**) with NIs (**2a-c**) in benzene solution, according to WB97XD/6-311G(d,p)(PCM)calculations ($\Delta$H and $\Delta$G are given in kcal/mol$^{-1}$; $\Delta$S are given in cal/mol$^{-1}$ K$^{-1}$).

| Reaction | Path | Transition | ΔH | ΔG | ΔS |
|---|---|---|---|---|---|
| **1 + 2a** | **A** | **1 + 2a → $MC_{4a}$** | -6.61 | 3.32 | -42.41 |
| | | **1 + 2a → $TS_{4a}$** | -0.74 | 11.52 | -50.26 |
| | | **1 + 2a → 4a** | -64.91 | -50.90 | -56.14 |
| | **B** | **1 + 2a → $MC_{5a}$** | -8.80 | 0.69 | -40.97 |
| | | **1 + 2a → $TS_{5a}$** | -2.25 | 10.50 | -51.90 |
| | | **1 + 2a → 5a** | -67.06 | -53.01 | -56.26 |
| **1 + 2b** | **A** | **1 + 2b → $MC_{4b}$** | -7.16 | 1.35 | -28.54 |
| | | **1 + 2b → $TS_{4b}$** | -0.13 | 13.38 | -45.31 |
| | | **1 + 2b → 4b** | -64.09 | -49.13 | -50.17 |
| | **B** | **1 + 2b → $MC_{5b}$** | -9.02 | 0.87 | -33.18 |
| | | **1 + 2b → $TS_{5b}$** | -2.43 | 10.73 | -44.13 |
| | | **1 + 2b → 5b** | -66.38 | -51.79 | -48.95 |
| **1 + 2c** | **A** | **1 + 2c → $MC_{4c}$** | -6.66 | 3.40 | -33.76 |
| | | **1 + 2c → $TS_{4c}$** | 0.28 | 13.54 | -44.48 |
| | | **1 + 2b → 4c** | -65.66 | -50.81 | -49.80 |
| | **B** | **1 + 2c → $MC_{5c}$** | -8.80 | 0.43 | -30.95 |
| | | **1 + 2c → $TS_{5c}$** | -2.27 | 11.11 | -44.89 |
| | | **1 + 2c → 5c** | -67.57 | -52.74 | -49.76 |

**Table S2.** The key parameters of the critical structures parameters for 32CA reaction of TNP (**1**) with NIs (**2a-c**) in benzene solution, according to WB97XD/6-311G(d,p)(PCM) calculations.

| | C3-C4 r [Å] | $l_{C3-C4}$ | C5-N1 r [Å] | $l_{C5-N1}$ | Δl | GEDT [e] |
|---|---|---|---|---|---|---|
| **$MC_{4a}$** | 3.260 | | 3.033 | | | 0.03 |
| **$TS_{4a}$** | 2.242 | 0.521 | 2.355 | 0.378 | 0.14 | 0.17 |
| **4a** | 1.516 | | 1.452 | | | |
| **$MC_{5a}$** | 3.333 | | 3.033 | | | 0.03 |
| **$TS_{5a}$** | 2.275 | 0.509 | 2.383 | 0.309 | 0.20 | 0.20 |
| **5a** | 1.526 | | 1.409 | | | |
| **$MC_{4b}$** | 3.161 | | 3.275 | | | 0.02 |
| **$TS_{4b}$** | 2.236 | 0.526 | 2.356 | 0.377 | 0.15 | 0.22 |
| **4b** | 1.517 | | 1.451 | | | |
| **$MC_{5b}$** | 3.358 | | 2.935 | | | 0.04 |
| **$TS_{5b}$** | 2.285 | 0.503 | 2.356 | 0.326 | 0.18 | 0.23 |
| **5b** | 1.527 | | 1.408 | | | |
| **$MC_{4c}$** | 3.263 | | 3.049 | | | 0.03 |
| **$TS_{4c}$** | 2.233 | 0.527 | 2.375 | 0.364 | 0.16 | 0.20 |
| **4c** | 1.515 | | 1.452 | | | |
| **$MC_{5c}$** | 3.361 | | 2.985 | | | 0.03 |
| **$TS_{5c}$** | 2.277 | 0.507 | 2.384 | 0.309 | 0.20 | 0.22 |
| **5c** | 1.525 | | 1.410 | | | |

**Table S3.** Thermochemistry and cartesian coordinates of **1** (WB97XD/6-311G(d,p)(PCM), benzene solution).

| | | |
|---|---|---|
| Zero-point correction = | 0.055027 (Hartree/Particle) | **1** |
| Thermal correction to Energy = | 0.062839 (Hartree/Particle) | |
| Thermal correction to Enthalpy = | 0.063783 (Hartree/Particle) | |
| Thermal correction to Gibbs Free Energy = | 0.020554 (Hartree/Particle) | |
| Sum of electronic and zero-point Energies = | -1701.121509 (Hartree/Particle) | |
| Sum of electronic and thermal Energies = | -1701.113697 (Hartree/Particle) | |
| Sum of electronic and thermal Enthalpies = | -1701.112753 (Hartree/Particle) | |
| Sum of electronic and thermal Free Energies = | -1701.155982 (Hartree/Particle) | |

| Center | Coordinates (Angstroms) | | |
|---|---|---|---|
| | X | Y | Z |
| C | 1.44414400 | 0.00034900 | -0.40887500 |
| H | 1.36398000 | 0.00236000 | -1.48885100 |
| C | 0.46029900 | -0.00120000 | 0.46738000 |
| H | 0.65856700 | -0.00315100 | 1.53270300 |
| C | -0.99526500 | -0.00016300 | 0.06194100 |
| Cl | -2.00686000 | -0.00526100 | 1.53303300 |
| Cl | -1.38237500 | 1.45888000 | -0.90248900 |
| Cl | -1.38201100 | -1.45263600 | -0.91240300 |
| N | 2.83611000 | -0.00028900 | 0.06239300 |
| O | 3.36166600 | 1.07914900 | 0.22442000 |
| O | 3.36093300 | -1.08012300 | 0.22412200 |

**Table S4.** Thermochemistry and cartesian coordinates of **2a** (WB97XD/6-311G(d,p)(PCM), benzene solution).

| | | |
|---|---|---|
| Zero-point correction = | 0.187141 (Hartree/Particle) | **2a** |
| Thermal correction to Energy = | 0.200514 (Hartree/Particle) | |
| Thermal correction to Enthalpy = | 0.201458 (Hartree/Particle) | |
| Thermal correction to Gibbs Free Energy = | 0.143588 (Hartree/Particle) | |
| Sum of electronic and zero-point Energies = | -3184.126598 (Hartree/Particle) | |
| Sum of electronic and thermal Energies = | -3184.113226 (Hartree/Particle) | |
| Sum of electronic and thermal Enthalpies = | -3184.112281 (Hartree/Particle) | |
| Sum of electronic and thermal Free Energies = | -3184.170151 (Hartree/Particle) | |

| Center | Coordinates (Angstroms) | | |
|---|---|---|---|
| | X | Y | Z |
| C | 2.64167900 | 0.72890700 | -0.16992300 |
| C | 3.91005700 | 0.09153900 | -0.06120900 |
| C | 6.19505900 | 0.11498700 | 0.69564800 |
| H | 4.80460800 | 1.72086100 | 1.02852800 |
| C | 4.96171600 | 0.73942400 | 0.59863200 |
| C | 5.33698700 | -1.79851500 | -0.49313400 |
| C | 4.10244900 | -1.18027400 | -0.61406700 |
| H | 5.48427200 | -2.78473200 | -0.91654200 |
| N | 1.61691900 | 1.27028900 | -0.19383500 |
| N | 0.56461500 | 1.96702000 | -0.25376600 |
| H | 7.01069100 | 0.61783100 | 1.20119400 |
| H | 7.34889700 | -1.63841800 | 0.24123800 |
| C | 6.38402700 | -1.15294900 | 0.15579200 |
| C | -0.63151300 | 1.25113500 | -0.15858600 |
| C | -1.81312800 | 1.99925000 | -0.24909800 |
| H | -1.73724200 | 3.07092000 | -0.38864000 |
| C | -1.96978500 | -0.75137300 | 0.10667500 |
| C | -3.12392900 | 0.01014400 | 0.01514800 |
| H | -3.95477800 | 1.97932300 | -0.23360200 |
| C | -0.72861600 | -0.13407600 | 0.02017000 |
| C | -3.05049000 | 1.38786100 | -0.16288200 |
| Br | -4.82671900 | -0.83505700 | 0.13956000 |
| H | 0.16549600 | -0.74186800 | 0.10012000 |
| H | -2.03153400 | -1.82325800 | 0.24822300 |
| H | 3.28693700 | -1.67116700 | -1.13088800 |

**Table S5.** Thermochemistry and cartesian coordinates of **$MC_{4a}$** (WB97XD/6-311G(d,p)(PCM), benzene solution).

| | | |
|---|---|---|
| Zero-point correction = | 0.244108 (Hartree/Particle) | **$MC_{4a}$** |
| Thermal correction to Energy = | 0.268530 (Hartree/Particle) | |
| Thermal correction to Enthalpy = | 0.269474 (Hartree/Particle) | |
| Thermal correction to Gibbs Free Energy = | 0.184190 (Hartree/Particle) | |
| Sum of electronic and zero-point Energies = | -4885.260931 (Hartree/Particle) | |
| Sum of electronic and thermal Energies = | -4885.236510 (Hartree/Particle) | |
| Sum of electronic and thermal Enthalpies = | -4885.235565 (Hartree/Particle) | |
| Sum of electronic and thermal Free Energies = | -4885.320850 (Hartree/Particle) | |

| **Center** | **Coordinates (Angstroms)** | | |
|---|---|---|---|
| | **X** | **Y** | **Z** |
| C | -1.98043300 | -1.40758700 | 0.49449400 |
| C | -2.38990200 | 1.72640600 | -0.30407200 |
| C | -1.36412200 | 2.24545900 | 0.35005200 |
| H | -2.49311100 | 1.59752100 | -1.37121000 |
| H | -1.39704400 | 2.34256300 | 1.42771500 |
| N | -1.02426100 | -0.91684900 | 0.93023600 |
| C | -3.13968200 | -2.13401500 | 0.09967100 |
| C | -3.05395200 | -3.05600300 | -0.95084800 |
| C | -4.35618500 | -1.91933900 | 0.75767400 |
| C | -4.18237400 | -3.76522200 | -1.32764700 |
| H | -2.10975300 | -3.21155800 | -1.45822600 |
| C | -5.47674300 | -2.63129200 | 0.35951100 |
| H | -4.41807600 | -1.19204200 | 1.55756700 |
| C | -5.39356500 | -3.55477800 | -0.67617900 |
| H | -4.11661500 | -4.48416000 | -2.13541400 |
| H | -6.42070600 | -2.46090200 | 0.86257500 |
| H | -6.27417500 | -4.10860600 | -0.97910700 |
| N | -0.06802000 | -0.26966600 | 1.44234800 |
| C | 1.20671900 | -0.64074800 | 0.99043100 |
| C | 1.45278800 | -1.52674700 | -0.06297500 |
| C | 2.29511800 | -0.06069000 | 1.65043300 |
| C | 2.75321200 | -1.81473100 | -0.45471900 |
| H | 0.63062900 | -1.99450500 | -0.59211100 |
| C | 3.59255500 | -0.34234900 | 1.26211300 |
| H | 2.10274100 | 0.61569700 | 2.47382000 |
| C | 3.81604500 | -1.21934200 | 0.20651600 |
| H | 2.93220000 | -2.50172800 | -1.27243200 |
| H | 4.42623600 | 0.11544800 | 1.77944900 |
| Br | 5.60007000 | -1.61263500 | -0.32897100 |
| N | -3.57785000 | 1.31207500 | 0.42968500 |
| O | -4.55390700 | 1.05257600 | -0.24600000 |
| O | -3.53046600 | 1.24183900 | 1.64427200 |
| C | -0.15275200 | 2.80845500 | -0.34568200 |
| Cl | -0.59944900 | 4.38368200 | -1.09193300 |
| Cl | 0.44161000 | 1.71117800 | -1.62239200 |
| Cl | 1.14258900 | 3.09604400 | 0.83942300 |

**Table S4.** Thermochemistry and cartesian coordinates of **$MC_{5a}$** (WB97XD/6-311G(d,p)(PCM), benzene solution).

| | | |
|---|---|---|
| Zero-point correction = | 0.244331 (Hartree/Particle) | **$MC_{5a}$** |
| Thermal correction to Energy = | 0.268731 (Hartree/Particle) | |
| Thermal correction to Enthalpy = | 0.269675 (Hartree/Particle) | |
| Thermal correction to Gibbs Free Energy = | 0.183704 (Hartree/Particle) | |
| Sum of electronic and zero-point Energies = | -4885.264406 (Hartree/Particle) | |
| Sum of electronic and thermal Energies = | -4885.240006 (Hartree/Particle) | |
| Sum of electronic and thermal Enthalpies = | -4885.239062 (Hartree/Particle) | |
| Sum of electronic and thermal Free Energies = | -4885.325033 (Hartree/Particle) | |

| **Center** | **Coordinates (Angstroms)** | | |
|---|---|---|---|
| | **X** | **Y** | **Z** |
| C | -1.02890200 | 1.53770500 | -0.84619900 |
| C | -2.10695200 | -1.21331200 | 0.69698100 |
| C | -0.88114400 | -1.70451900 | 0.68129500 |
| H | -2.32274000 | -0.30367400 | 1.24612300 |
| H | -0.53193100 | -2.59738500 | 0.18850700 |
| N | -0.19985500 | 0.77716600 | -1.12233400 |
| C | -2.05871100 | 2.40411000 | -0.37743400 |
| C | -2.20407300 | 2.61370200 | 0.99943700 |
| C | -2.92914500 | 3.01760700 | -1.28440500 |
| C | -3.21900000 | 3.43880300 | 1.45837500 |
| H | -1.52319600 | 2.12671900 | 1.68715700 |
| C | -3.94903100 | 3.82664300 | -0.80900700 |
| H | -2.80226700 | 2.85171100 | -2.34703400 |
| C | -4.09327900 | 4.03920800 | 0.55822800 |
| H | -3.33360500 | 3.60668700 | 2.52237700 |
| H | -4.63028800 | 4.29708900 | -1.50754800 |
| H | -4.88934000 | 4.67736000 | 0.92336900 |
| N | 0.59045800 | -0.14964300 | -1.46708500 |
| C | 1.91629300 | 0.00253900 | -1.03722500 |
| C | 2.37250400 | 1.01973800 | -0.19341000 |
| C | 2.82208600 | -0.96978600 | -1.47577600 |
| C | 3.70403000 | 1.06460800 | 0.19793300 |
| H | 1.68974000 | 1.77633500 | 0.17428000 |
| C | 4.14921100 | -0.92703800 | -1.08951400 |
| H | 2.46371600 | -1.75796300 | -2.12695200 |
| C | 4.58466000 | 0.09445500 | -0.25255800 |
| H | 4.04643200 | 1.85250900 | 0.85699500 |
| H | 4.84185600 | -1.68345200 | -1.43651600 |
| Br | 6.41162400 | 0.15632100 | 0.28230400 |
| C | -3.28649700 | -1.81956700 | 0.00577600 |
| Cl | -4.52227100 | -2.18654700 | 1.25145100 |
| Cl | -2.89873800 | -3.30213500 | -0.89455600 |
| Cl | -3.95400800 | -0.60103000 | -1.12689000 |
| N | 0.17101000 | -1.03044000 | 1.43830200 |
| O | -0.04442000 | 0.08300200 | 1.88670400 |
| O | 1.20398200 | -1.64813100 | 1.57471500 |

**Table S5.** Thermochemistry and cartesian coordinates of $\mathbf{TS_{4a}}$ (WB97XD/6-311G(d,p)(PCM), benzene solution).

| | | |
|---|---|---|
| Zero-point correction = | 0.243937 (Hartree/Particle) | $\mathbf{TS_{4a}}$ |
| Thermal correction to Energy = | 0.267075 (Hartree/Particle) | |
| Thermal correction to Enthalpy = | 0.268019 (Hartree/Particle) | |
| Thermal correction to Gibbs Free Energy = | 0.186460 (Hartree/Particle) | |
| Sum of electronic and zero-point Energies = | -4885.250300 (Hartree/Particle) | |
| Sum of electronic and thermal Energies = | -4885.227163 (Hartree/Particle) | |
| Sum of electronic and thermal Enthalpies = | -4885.226219 (Hartree/Particle) | |
| Sum of electronic and thermal Free Energies = | -4885.307778 (Hartree/Particle) | |

| **Center** | **Coordinates (Angstroms)** | | |
|---|---|---|---|
| | **X** | **Y** | **Z** |
| C | -2.05554600 | -0.99320300 | -0.06993300 |
| C | -2.31935300 | 1.21747200 | 0.19172600 |
| C | -1.10649200 | 1.66677200 | 0.63031700 |
| H | -2.71011700 | 1.38372300 | -0.80076000 |
| H | -0.94257600 | 1.84554600 | 1.68317100 |
| N | -0.96164300 | -1.07472300 | 0.42122700 |
| C | -3.27358200 | -1.73337800 | -0.27545100 |
| C | -4.04159000 | -1.48363700 | -1.41598000 |
| C | -3.71158400 | -2.65607000 | 0.68225200 |
| C | -5.23170400 | -2.16734200 | -1.60789800 |
| H | -3.70258800 | -0.75434800 | -2.14320900 |
| C | -4.90694800 | -3.32985800 | 0.48504600 |
| H | -3.11756600 | -2.83112300 | 1.57211900 |
| C | -5.66411500 | -3.08708400 | -0.65760300 |
| H | -5.82690000 | -1.97721600 | -2.49370200 |
| H | -5.25113100 | -4.04271800 | 1.22583900 |
| H | -6.60120300 | -3.61329300 | -0.80428500 |
| N | -0.07997800 | -0.41288900 | 1.03778800 |
| C | 1.26668600 | -0.67718000 | 0.69322200 |
| C | 1.65486400 | -1.28932900 | -0.49809300 |
| C | 2.23527000 | -0.27543000 | 1.61054900 |
| C | 2.99975600 | -1.49040200 | -0.77297800 |
| H | 0.90701500 | -1.61292600 | -1.21364100 |
| C | 3.58084100 | -0.46752500 | 1.34023000 |
| H | 1.92288900 | 0.19261700 | 2.53709600 |
| C | 3.95377300 | -1.07228300 | 0.14587300 |
| H | 3.29936100 | -1.96680200 | -1.69884700 |
| H | 4.33049100 | -0.15281800 | 2.05609600 |
| Br | 5.79674100 | -1.34498300 | -0.22987100 |
| N | -3.38294600 | 1.05086700 | 1.17202500 |
| O | -4.52337700 | 1.10692200 | 0.74777000 |
| O | -3.08600400 | 0.84904300 | 2.33602300 |
| C | -0.20421600 | 2.41143100 | -0.31312000 |
| Cl | -0.93814300 | 4.02962000 | -0.66190600 |
| Cl | -0.00917700 | 1.54598500 | -1.86926000 |
| Cl | 1.39420300 | 2.69354300 | 0.41522700 |

**Table S6.** Thermochemistry and cartesian coordinates of **TS$_{5a}$** (WB97XD/6-311G(d,p)(PCM), benzene solution).

| | | |
|---|---|---|
| Zero-point correction = | 0.244003 (Hartree/Particle) | **TS$_{5a}$** |
| Thermal correction to Energy = | 0.266957 (Hartree/Particle) | |
| Thermal correction to Enthalpy = | 0.267901 (Hartree/Particle) | |
| Thermal correction to Gibbs Free Energy = | 0.187125 (Hartree/Particle) | |
| Sum of electronic and zero-point Energies = | -4885.252518 (Hartree/Particle) | |
| Sum of electronic and thermal Energies = | -4885.229564 (Hartree/Particle) | |
| Sum of electronic and thermal Enthalpies = | -4885.228620 (Hartree/Particle) | |
| Sum of electronic and thermal Free Energies = | -4885.309397 (Hartree/Particle) | |

| **Center** | **Coordinates (Angstroms)** | | |
|---|---|---|---|
| | **X** | **Y** | **Z** |
| C | -1.41878700 | -0.98003100 | 0.13129700 |
| C | -1.71894000 | 1.14010600 | -0.63808500 |
| C | -0.53411400 | 1.71460200 | -0.28432700 |
| H | -1.83309800 | 0.75178500 | -1.64139200 |
| H | -0.38674900 | 2.44280300 | 0.49587600 |
| N | -0.39497800 | -0.79838800 | 0.73300900 |
| C | -2.47437300 | -1.95945400 | 0.01475100 |
| C | -2.87105800 | -2.38523100 | -1.25407800 |
| C | -3.11165300 | -2.44850700 | 1.15801700 |
| C | -3.89217700 | -3.31431400 | -1.37630700 |
| H | -2.37813900 | -1.98558700 | -2.13245800 |
| C | -4.14191500 | -3.36616600 | 1.02417200 |
| H | -2.80151000 | -2.10097300 | 2.13604400 |
| C | -4.53032400 | -3.79970600 | -0.23942900 |
| H | -4.19645600 | -3.65467700 | -2.35870500 |
| H | -4.64118300 | -3.74620900 | 1.90726300 |
| H | -5.33428900 | -4.51970300 | -0.33829600 |
| N | 0.42573000 | 0.06431900 | 1.14168100 |
| C | 1.78183600 | -0.14475600 | 0.81580500 |
| C | 2.22773500 | -1.13191700 | -0.06447400 |
| C | 2.69475300 | 0.73832000 | 1.39096400 |
| C | 3.57862400 | -1.24419300 | -0.35152000 |
| H | 1.52249600 | -1.81730500 | -0.51852900 |
| C | 4.04463000 | 0.63384400 | 1.10162600 |
| H | 2.33255200 | 1.50477300 | 2.06469900 |
| C | 4.47633400 | -0.36049600 | 0.23322700 |
| H | 3.92751800 | -2.01114200 | -1.03076400 |
| H | 4.75282200 | 1.32093000 | 1.54585100 |
| Br | 6.32843100 | -0.51702900 | -0.15941100 |
| C | -2.99106300 | 1.60020400 | 0.03133200 |
| Cl | -3.29349800 | 3.33612800 | -0.35629700 |
| Cl | -2.88782900 | 1.43818600 | 1.81323400 |
| Cl | -4.39348200 | 0.66917200 | -0.56383100 |
| N | 0.57473400 | 1.61345600 | -1.19448200 |
| O | 0.52894500 | 0.76919300 | -2.07838600 |
| O | 1.51571200 | 2.36361900 | -1.00314900 |

**Table S7.** Thermochemistry and cartesian coordinates of **4a** (WB97XD/6-311G(d,p)(PCM), benzene solution).

| | | |
|---|---|---|
| Zero-point correction = | 0.249958 (Hartree/Particle) | **4a** |
| Thermal correction to Energy = | 0.272038 (Hartree/Particle) | |
| Thermal correction to Enthalpy = | 0.272982 (Hartree/Particle) | |
| Thermal correction to Gibbs Free Energy = | 0.194221 (Hartree/Particle) | |
| Sum of electronic and zero-point Energies = | -4885.351504 (Hartree/Particle) | |
| Sum of electronic and thermal Energies = | -4885.329424 (Hartree/Particle) | |
| Sum of electronic and thermal Enthalpies = | -4885.328479 (Hartree/Particle) | |
| Sum of electronic and thermal Free Energies = | -4885.407241 (Hartree/Particle) | |

| **Center** | **Coordinates (Angstroms)** | | |
|---|---|---|---|
| | **X** | **Y** | **Z** |
| C | 2.01955700 | -0.77644800 | 0.03984400 |
| C | 2.10379900 | 0.55547100 | -0.67926200 |
| C | 0.72935500 | 1.16291900 | -0.37711200 |
| H | 2.94560300 | 1.17538800 | -0.38328700 |
| H | 0.24974900 | 1.50041500 | -1.29629600 |
| N | 0.84284600 | -0.99751600 | 0.48830000 |
| C | 3.13471000 | -1.71081400 | 0.21854200 |
| C | 4.42854600 | -1.37186500 | -0.18182500 |
| C | 2.90673200 | -2.95992100 | 0.80773600 |
| C | 5.47494600 | -2.26746900 | 0.00216100 |
| H | 4.62893400 | -0.41607000 | -0.65072700 |
| C | 3.95311800 | -3.84719800 | 0.98863400 |
| H | 1.90290200 | -3.22200900 | 1.11843700 |
| C | 5.24193400 | -3.50414100 | 0.58673000 |
| H | 6.47446500 | -1.99445200 | -0.31508500 |
| H | 3.76600900 | -4.81204900 | 1.44523300 |
| H | 6.05876900 | -4.20227500 | 0.72822200 |
| N | 0.01701000 | 0.06238800 | 0.24653200 |
| C | -1.36147400 | -0.20824100 | 0.12020000 |
| C | -1.91238400 | -1.25718900 | 0.85983200 |
| C | -2.18607100 | 0.54267000 | -0.71403400 |
| C | -3.26305400 | -1.54563500 | 0.76706600 |
| H | -1.27432700 | -1.84864800 | 1.50143900 |
| C | -3.54280400 | 0.25943800 | -0.80071600 |
| H | -1.79792700 | 1.35853200 | -1.30818900 |
| C | -4.07607400 | -0.78221000 | -0.06056900 |
| H | -3.67931900 | -2.36106500 | 1.34476900 |
| H | -4.17272300 | 0.85246700 | -1.45111400 |
| Br | -5.93339300 | -1.17160000 | -0.17793900 |
| N | 2.28063600 | 0.32436600 | -2.16914700 |
| O | 3.38029800 | 0.53039800 | -2.62943200 |
| O | 1.31363300 | -0.06360500 | -2.78611600 |
| C | 0.84677700 | 2.39711800 | 0.55714400 |
| Cl | 1.73569700 | 3.67386800 | -0.33296200 |
| Cl | 1.73289800 | 1.98181100 | 2.04873500 |
| Cl | -0.76000500 | 3.01478200 | 0.99321600 |

**Table S8.** Thermochemistry and cartesian coordinates of **5a** (WB97XD/6-311G(d,p)(PCM), benzene solution).

| | | |
|---|---|---|
| Zero-point correction = | 0.249742 (Hartree/Particle) | **5a** |
| Thermal correction to Energy = | 0.271856 (Hartree/Particle) | |
| Thermal correction to Enthalpy = | 0.272800 (Hartree/Particle) | |
| Thermal correction to Gibbs Free Energy = | 0.194095 (Hartree/Particle) | |
| Sum of electronic and zero-point Energies = | -4885.354958 (Hartree/Particle) | |
| Sum of electronic and thermal Energies = | -4885.332844 (Hartree/Particle) | |
| Sum of electronic and thermal Enthalpies = | -4885.331900 (Hartree/Particle) | |
| Sum of electronic and thermal Free Energies = | -4885.410605 (Hartree/Particle) | |

| **Center** | **Coordinates (Angstroms)** | | |
|---|---|---|---|
| | **X** | **Y** | **Z** |
| C | 1.78744600 | 0.67450800 | -0.37122200 |
| C | 1.86674800 | -0.84918700 | -0.38436700 |
| C | 0.43316600 | -1.17481900 | -0.81240400 |
| H | 2.57947000 | -1.24747700 | -1.10660400 |
| H | -0.00868300 | -2.05304300 | -0.34370100 |
| N | 0.57829000 | 1.09108000 | -0.44451000 |
| C | 2.91819500 | 1.60966200 | -0.31533900 |
| C | 4.13435700 | 1.29625600 | -0.92381700 |
| C | 2.76389800 | 2.84815900 | 0.31110000 |
| C | 5.17922700 | 2.20899900 | -0.90844900 |
| H | 4.26874000 | 0.34171500 | -1.41987400 |
| C | 3.81152000 | 3.75590900 | 0.32785300 |
| H | 1.82029000 | 3.08582500 | 0.78695700 |
| C | 5.02092200 | 3.43936700 | -0.28195500 |
| H | 6.11773000 | 1.95986900 | -1.38940100 |
| H | 3.68534200 | 4.71270500 | 0.82081200 |
| H | 5.83811300 | 4.15113900 | -0.26814800 |
| N | -0.26856100 | 0.02365100 | -0.57551800 |
| C | -1.64405800 | 0.16643200 | -0.36854000 |
| C | -2.14706400 | 1.35304800 | 0.16583600 |
| C | -2.51847800 | -0.87615500 | -0.67437100 |
| C | -3.50677300 | 1.49225500 | 0.39213800 |
| H | -1.46871800 | 2.16149300 | 0.40005500 |
| C | -3.87776400 | -0.74007800 | -0.43367500 |
| H | -2.16122600 | -1.79287600 | -1.12558300 |
| C | -4.36688100 | 0.44336500 | 0.09748900 |
| H | -3.89062300 | 2.41581800 | 0.80625300 |
| H | -4.54987300 | -1.55314600 | -0.67614300 |
| Br | -6.23153700 | 0.63384000 | 0.41791800 |
| C | 2.22829100 | -1.48018600 | 0.98008300 |
| Cl | 2.12437600 | -3.26364900 | 0.83933500 |
| Cl | 1.10253000 | -0.93131500 | 2.25374000 |
| Cl | 3.89133300 | -1.05592700 | 1.44903200 |
| N | 0.44376300 | -1.54251000 | -2.33240500 |
| O | -0.33145600 | -0.97179400 | -3.05581400 |
| O | 1.21798000 | -2.41721100 | -2.65202200 |
